# Oral topotecan: bioavailability and effect of food co-administration

**DOI:** 10.1038/sj.bjc.6690532

**Published:** 1999-07

**Authors:** V M M Herben, H Rosing, W W ten Bokkel Huinink, D M van Zomeren, D Batchelor, E Doyle, F D Beusenberg, J H Beijnen, J H M Schellens

**Affiliations:** Department of Pharmacy and Pharmacology, Slotervaart Hospital/The Netherlands Cancer Institute, Amsterdam, The Netherlands; Department of Medical Oncology, Antoni van Leeuwenhoek Hospital/The Netherlands Cancer Institute, Amsterdam, The Netherlands; Department of Drug Metabolism and Pharmacokinetics, SmithKline Beecham Pharmaceuticals, Hertfordshire, UK; SmithKline Beecham Farma, Rijswijk, The Netherlands

**Keywords:** topotecan, oral, bioavailability, food

## Abstract

The aims of the study were twofold: (1) to evaluate the effect of food on the relative oral bioavailability of topotecan gelatin capsules in patients with solid tumours, and (2) to determine the absolute bioavailability of oral topotecan with reference to the intravenous (i.v.) formulation. The study had a randomized two-period cross-over design. On day 1 of the first treatment course patients were administered 2.3 mg m^−2^ day^−1^ of oral topotecan with or without a high-fat breakfast. They crossed over to receive the alternate regimen on day 2. In the second course (3 weeks later) fasted patients received topotecan orally (2.3 mg m^−2^ day^−1^) or i.v. (1.5 mg m^−3^ day). They crossed over to receive the alternate regimen on day 2. On days 3–5 of both treatment courses patients received oral topotecan. Plasma pharmacokinetics were performed on days 1 and 2 of the first and second course using a high-performance liquid chromatographic assay. Eighteen patients were enrolled in the study. The ratio of the area under the curve to infinity during fasted and high-fat treatment was 0.93 ± 0.23 (90% confidence interval (CI) 0.83–1.03). Maximal plasma concentrations of topotecan were similar after ingestion of the capsules with (10.6 ± 4.4 ng ml^−1^) or without food (9.2 ± 4.1 ng ml^−1^) (*P* = 0.130). The time needed to reach maximal plasma levels was significantly prolonged after food intake (median 3.1 h, range 2.8–6.1) compared to fasted conditions (2.0 h, range 1.1–8.1) (*P* = 0.013). The absolute bioavailability of topotecan averaged 42 ± 13% (90% CI 37– 47%). The apparent terminal half-life was significantly longer after administration of oral topotecan (3.9 ± 1.0 h) than after i.v. administration (2.7 ± 0.4 h) (*P* < 0.001). Topotecan demonstrates suitable bioavailability for oral treatment. Co-administration of the topotecan gelatin capsules with a high-fat breakfast leads to a small decrease in absorption rate but does not affect the extent of absorption. © 1999 Cancer Research Campaign


*British Journal of Cancer* (1999) **80**(9), 1380–1386
© 1999 Cancer Research Campaign
Article no. bjoc.1999.0532

### Oral topotecan: bioavailability and effect of food co-administration

**VMM Herben[1,2], H Rosing[1], WW ten Bokkel Huinink[2], DM van Zomeren[1], D Batchelor[2], E Doyle[3], FD Beusenberg[4], JH Beijnen[1,2] and JHM Schellens[2]**

[1]Slotervaart Hospital/The Netherlands Cancer Institute, Department of Pharmacy and Pharmacology, Amsterdam, The Netherlands; [2]Antoni van Leeuwenhoek Hospital/The Netherlands Cancer Institute, Department of Medical Oncology, Amsterdam, The Netherlands; [3]SmithKline Beecham Pharmaceuticals, Department of Drug Metabolism and Pharmacokinetics, Hertfordshire, UK; [4]SmithKline Beecham Farma, Rijswijk, The Netherlands

**Summary** The aims of the study were twofold: (1) to evaluate the effect of food on the relative oral bioavailability of topotecan gelatin capsules in patients with solid tumours, and (2) to determine the absolute bioavailability of oral topotecan with reference to the intravenous (i.v.) formulation. The study had a randomized two-period cross-over design. On day 1 of the first treatment course patients were administered 2.3 mg $m^{-2}$ $day^{-1}$ of oral topotecan with or without a high-fat breakfast. They crossed over to receive the alternate regimen on day 2. In the second course (3 weeks later) fasted patients received topotecan orally (2.3 mg $m^{-2}$ $day^{-1}$) or i.v. (1.5 mg $m^{-3}$ day). They crossed over to receive the alternate regimen on day 2. On days 3–5 of both treatment courses patients received oral topotecan. Plasma pharmacokinetics were performed on days 1 and 2 of the first and second course using a high-performance liquid chromatographic assay. Eighteen patients were enrolled in the study. The ratio of the area under the curve to infinity during fasted and high-fat treatment was 0.93 ± 0.23 (90% confidence interval (CI) 0.83–1.03). Maximal plasma concentrations of topotecan were similar after ingestion of the capsules with (10.6 ± 4.4 ng $ml^{-1}$) or without food (9.2 ± 4.1 ng $ml^{-1}$) ($P = 0.130$). The time needed to reach maximal plasma levels was significantly prolonged after food intake (median 3.1 h, range 2.8–6.1) compared to fasted conditions (2.0 h, range 1.1–8.1) ($P = 0.013$). The absolute bioavailability of topotecan averaged 42 ± 13% (90% CI 37– 47%). The apparent terminal half-life was significantly longer after administration of oral topotecan (3.9 ± 1.0 h) than after i.v. administration (2.7 ± 0.4 h) ($P < 0.001$). Topotecan demonstrates suitable bioavailability for oral treatment. Co-administration of the topotecan gelatin capsules with a high-fat breakfast leads to a small decrease in absorption rate but does not affect the extent of absorption.

**Keywords:** topotecan; oral; bioavailability; food

Topotecan (Hycamtin) is one of the prototypes of a promising new class of cytotoxic drugs that act through inhibition of topoisomerase I. Topoisomerase I is a nuclear enzyme that relieves the torsional strain in DNA ahead of the moving replication fork (Hsiang et al, 1988, 1989). Inhibition of the enzyme results in lethal DNA damage during transcription and replication. The E-ring lactone in the basic structure of topotecan is considered to be essential for interaction with the topoisomerase I enzyme (Hertzberg et al, 1989; Pommier et al, 1991). This lactone moiety is quite labile and under physiological conditions topotecan exists in a dynamic, pH-dependent equilibrium with an inactive carboxylate form (Figure 1) (Underberg et al, 1990).

Topotecan is a cell cycle-specific drug for the S phase and exhibits schedule-dependent cytotoxicity in vitro as well as in vivo in preclinical tumour models. Enhanced activity was observed with repeated or prolonged drug administration (Burris et al, 1992; Supko et al, 1992; Houghton et al, 1995). Prolonged administration increases the probability that tumour cells will be exposed to topotecan during DNA replication, i.e. when they are most susceptible. In-vivo in patients there is yet no convincing evidence of a clinically relevant schedule dependency.

Intravenous (i.v.) topotecan administered as daily 30-min infusions for 5 days has recently received marketing approval for the treatment of patients with ovarian cancer after failure of initial or subsequent platinum-based chemotherapy. An oral gelatin capsule formulation has now been developed (Creemers et al, 1997; Gerrits et al 1998). A phase I study has determined the maximum tolerated dose (MTD) for oral topotecan to be 2.3 mg $m^{-2}$ for the daily times five dosing schedule (Gerrits et al, 1998).

In previous studies, bioavailability of the i.v. formulation of topotecan administered orally was 30–44% with moderate interpatient variability (Eckardt et al, 1995; Schellens et al, 1996). In these studies, bioavailability was tested in fasted patients. This situation does not, however, reflect the true clinical setting. Food can have marked effects on drug absorption by increasing, decreasing, or sometimes simply delaying it. Food can also influence drug metabolism, and certain food components, such as protein and fat, are particularly potent in this respect. For an oral formulation it is therefore essential to investigate the effect of food co-administration on the pharmacokinetics.

The aims of the present study were (1) to evaluate the effect of fed and fasted conditions on the bioavailability and pharmacokinetics of oral topotecan, and (2) to determine the absolute bioavailability of the gelatin capsule formulation.

*Received 10 June 1998*
*Revised 8 December 1998*
*Accepted 21 January 1999*

*Correspondence to:* VMM Herben, Slotervaart Hospital/The Netherlands Cancer Institute, Department of Pharmacy and Pharmacology, Louwesweg 6, 1066 EC Amsterdam, The Netherlands

**Table 1** Mean (± s.d.) pharmacokinetic parameters of topotecan after oral administration with and without food

| Parameter | Fasted | Fed | Ratio | 90% CI |
|---|---|---|---|---|
| $AUC_{0-t}$ (h ng ml$^{-1}$) | | | | |
| Lactone | 19.6 ± 7.4 | 22.7 ± 6.7 | 0.86 ± 0.18 | 0.80–0.94 |
| Total | 57.9 ± 25.7 | 62.4 ± 24.4 | 0.93 ± 0.25 | 0.83–1.04 |
| $AUC_{0-\infty}$ (h ng ml$^{-1}$) | | | | |
| Lactone | 22.3 ± 8.3 | 25.4 ± 7.5 | 0.88 ± 0.17 | 0.81–0.95 |
| Total | 68.4 ± 29.8 | 73.7 ± 28.6 | 0.93 ± 0.23 | 0.83–1.03 |
| $C_{max}$ (ng ml$^{-1}$) | | | | |
| Lactone | 5.1 ± 2.6 | 5.1 ± 2.0 | 1.01 ± 0.35 | 0.87–1.17 |
| Total | 9.2 ± 4.1 | 10.6 ± 4.4 | 0.87 ± 0.31 | 0.74–1.01 |
| $t_{1/2}$ (h) | | | | |
| Lactone | 3.84 ± 0.78 | 3.35 ± 0.88 | 1.15 ± 0.18 | 1.06–1.24 |
| Total | 3.74 ± 0.95 | 3.49 ± 0.78 | 1.07 ± 0.22 | 0.98–1.18 |
| Urinary excretion (%) total | 28.2 ± 13.3 | 31.6 ± 16.1 | NC | NC |

NC, not calculated because of carry-over effects between treatments.

### METHODS

### Study design

The study had an open label randomized two-period cross-over design (Figure 2). In the first treatment course the effect of food co-administration on the bioavailability of topotecan was determined in fasted and non-fasted patients. Eighteen patients were randomized to the sequence of fed/fasted using the method of randomly permuted blocks. In the second treatment course, the absolute bioavailability of oral topotecan with reference to the i.v. formulation was determined in fasted patients only.

### Patient population

Patients were eligible for the study if they had a histologically confirmed malignant solid tumour, refractory to conventional therapy, or for which no standard effective therapy exists. Other eligibility criteria included an initial Eastern Cooperative Oncology Group (ECOG) performance status ≤2, anticipated life expectancy of ≥2 months and age ≥18 years. Previous anticancer chemotherapy had to be discontinued for at least 3 weeks before entry into the study, or 6 weeks in case of pretreatment with a nitrosourea or mitomycin C. All patients had to have acceptable bone marrow function, defined as white blood cell count (WBC) ≥3000 μl$^{-1}$, absolute neutrophil count (ANC) ≥1500 μl$^{-1}$, platelets ≥100 000 μl$^{-1}$ and haemoglobin ≥9.0 g dl$^{-1}$ (5.5 mmol l$^{-1}$); serum bilirubin ≤2 mg dl$^{-1}$ (34 μmol l$^{-1}$) and other liver function tests ≤ twice the normal upper limit or ≤5 times when related to liver metastases; adequate renal function, defined as serum creatinine ≤1.5 mg dl$^{-1}$ (120 μmol l$^{-1}$) or creatinine clearance ≥60 ml min$^{-1}$. Patients were excluded if they had leukaemia, gross ascites, uncontrolled infection, evidence of an active peptic ulcer or other documented gastrointestinal (GI) diseases that might influence GI motility, or if they were taking hydrogen antagonists, proton pump inhibitors or antacids. The study protocol was approved by the Medical Ethics Committee of the hospital. All patients gave written informed consent.

### Drug administration

Oral topotecan was supplied by SmithKline Beecham Pharmaceuticals (King of Prussia, PA, USA) as gelatin capsules containing 0.25 or 1.00 mg of the anhydrous free base. The oral dose was 2.3 mg m$^{-2}$ day$^{-1}$ for 5 consecutive days. The calculated daily dose was rounded to the nearest 0.25 mg. Intravenous topotecan was supplied as a lyophilized light yellow powder in vials containing 4 mg of topotecan as the free base, 48 mg of mannitol, 20 mg of tartaric acid and hydrochloric acid–sodium hydroxide for pH adjustment to 3.0. The content of each vial was reconstituted with 4 ml sterile water for injection prior to dilution in 50 ml of 0.9% sodium chloride and was administered i.v. over 30 min by a syringe pump. The i.v. dose was 1.5 mg m$^{-2}$ day$^{-1}$. In the second treatment course, the daily doses of oral and i.v. topotecan were reduced by 0.4 mg m$^{-2}$ and 0.25 mg m$^{-2}$, respectively, if during the first course patients had experienced (1) grade 4 neutropenia (i.e. ANC <500 μl$^{-1}$) associated with fever/infection or lasting ≥5 days; (2) grade 3 neutropenia (i.e. ANC 500–900 μL$^{-1}$) lasting beyond day 21 of the first treatment course; or (3) grade 4 thrombocytopenia (i.e. platelets <25 000 μl$^{-1}$). Following course 2, the daily dose of oral topotecan could be increased by 0.4 mg m$^{-2}$ day$^{-1}$, if during the previous course there was no toxicity greater than grade 2.

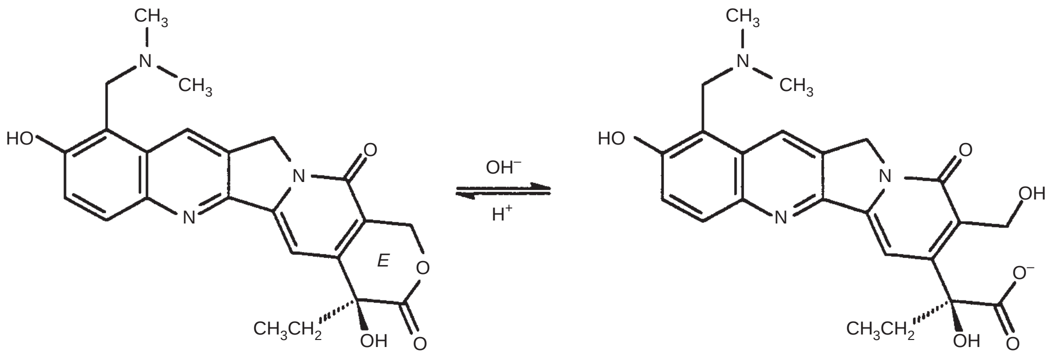

**Figure 1** Schematic representation of the reversible hydrolysis of the lactone ring (**A**) into the ring-opened carboxylate form (**B**) of topotecan

On day 1 of the first course, ‘non-fasting’ patients were fasted from midnight prior to dosing and received a high-fat breakfast, comprising two slices of toast, two fried eggs, one slice of ham, one slice of cheese, 20 g butter, 300 ml of milk, one glass of orange juice and one cup of tea/coffee with sugar. Oral topotecan was administered with 200 ml of water within 15 min of finishing breakfast. Fasted patients received oral topotecan with 200 ml of water after having fasted from midnight. For both treatment groups, food and fluid intake was restricted for 4 h after ingestion of topotecan. After day 1, patients crossed over to receive the alternate regimen on day 2. For the remaining 3 days, patients received topotecan capsules and were instructed to take them early in the morning at least 30 min prior to food.

Course 2 commenced 21 days after day 1 of course 1. In the second course, patients who were fed on day 1 of course 1 received i.v. topotecan on day 1. Patients who were fasted on day 1 of course 1 received oral topotecan on day 1. After day 1, patients crossed over to receive the alternate regimen on day 2. All patients were fasted from midnight the evening prior to dosing and were fed a light breakfast 30 min after dosing. For days 3–5, patients received oral topotecan.

### Blood sample collection

Blood samples (5 ml each), taken from an indwelling i.v. cannula, which in case of i.v. drug administration was placed in the arm contralateral to the arm receiving topotecan, were collected in heparinized tubes and immediately immersed into icewater. During i.v. administration, samples were obtained preinfusion, at 5, 15, 30, 45 and 60 min and 2, 3, 4, 6 and 8 h after the start of the infusion; for oral administration time-points were pre-dosing, 30, 60 and 90 min and 2, 3, 4, 6, 8, 10 and 12 h after ingestion. Plasma was obtained by immediate refrigerated centrifugation of the samples (5 min; 2500 ***g***). Plasma protein precipitation was performed by adding 1.0 ml of the separated plasma to 2.0 ml of cold methanol (–20°C) and vortex-mixing for 10 s. The sample was centrifuged at 3000 ***g*** for 5 min and the clear supernatant was transferred to a polypropylene tube and immediately stored at –70°C until analysis. The remaining plasma was stored at –30°C until analysis. Urine was collected over 24 h post-dosing and aliquots were stored at –30°C until analysis.

### Chemicals

For high-performance liquid chromatographic (HPLC) analysis, pure reference standards of topotecan (hydrochloride salt, SK&B 104864) and *N*-desmethyltopotecan (hydrochloride salt, SB 209780) were obtained from SmithKline Beecham Pharmaceuticals (King of Prussia, PA, USA). Solvents and reagents were of the highest purity available.

### HPLC analysis

For treatment course 1, plasma and urine samples were assayed for topotecan as the lactone form and as the total of lactone and carboxylate forms using a previously published method (Rosing et al, 1995). For course 2, plasma concentrations of topotecan and its metabolite *N*-desmethyltopotecan were determined using a newly developed and validated method. For the quantification of the lactone forms, plasma methanol extracts were diluted with distilled water (50:50 v/v) and stored at –30°C prior to analysis. An aliquot of 100 µl was injected onto the HPLC column (Zorbax SB-$C_{18}$ analytical column, 3.5 µm particle size, 4.6 × 75 mm internal diameter; Rockland Technologies Inc., Newport, DE, USA). The mobile phase consisted of a mixture of 75 mmol $l^{-1}$ $KH_2PO_4$ and triethylamine (adjusted to pH 6.0 with 4 mol $l^{-1}$ hydrochloric acid) with methanol (72.6:0.2:27.2 v/v/v). The operating temperature was 50°C and the flow-rate was 1.0 mL $min^{-1}$. For the determination of topotecan and *N*-desmethyltopotecan plasma levels as the total of their lactone plus ring-opened carboxylate forms, 100 µl of plasma were deproteinized with 200 µl of methanol. After vortex-mixing and centrifugation (23 000 ***g***, 3 min), an aliquot of the supernatant was diluted with phosphoric acid 25 mmol $l^{-1}$ (50:50 v/v). For the determination of

**Table 2** Mean (± s.d.) pharmacokinetic parameters of topotecan and *N*-desmethyltopotecan after oral and i.v administration

| | Topotecan | | | | N-desmethyltopotecan | | | |
|---|---|---|---|---|---|---|---|---|
| Parameter | Oral | i.v. | Ratio[a] | 90% CI[a] | Oral | i.v. | Ratio | 90% CI |
| $AUC_{0-t}$ (h ng $ml^{-1}$) | | | | | | | | |
| Lactone | 20.4 ± 8.0 | 35.9 ± 8.6 | 0.37 ± 0.12 | 0.32–0.42 | ND | ND | | |
| Total | 56.6 ± 23.8 | 91.2 ± 20.1 | 0.40 ± 0.14 | 0.35–0.46 | 2.7 ± 1.5 | 2.4 ± 1.4 | 0.71 ± 0.39 | 0.56–0.87 |
| $AUC_{0-\infty}$ (h ng $ml^{-1}$) | | | | | | | | |
| Lactone | 22.7 ± 9.5 | 38.6 ± 9.6 | 0.38 ± 0.13 | 0.33–0.44 | ND | ND | | |
| Total | 66.8 ± 29.4 | 103.8 ± 26.1 | 0.42 ± 0.13 | 0.37–0.47 | 4.6 ± 2.3 | 4.8 ± 2.6 | 0.62 ± 0.20 | 0.54–0.71 |
| $C_{max}$ (ng $ml^{-1}$) | | | | | | | | |
| Lactone | 6.3 ± 3.8 | 33.9 ± 8.1 | 0.12 ± 0.07 | 0.09–0.15 | ND | ND | | |
| Total | 10.2 ± 5.2 | 42.0 ± 8.8 | 0.16 ± 0.07 | 0.13–0.19 | 0.34 ± 0.15 | 0.44 ± 0.18 | 0.51 ± 0.19 | 0.43–0.60 |
| $t_{1/2}$ (h) | | | | | | | | |
| Lactone | 3.49 ± 0.87 | 2.64 ± 0.34 | 1.32 ± 0.35 | 1.18–1.48 | ND | ND | | |
| Total | 3.91 ± 1.00 | 2.74 ± 0.40 | 1.43 ± 0.29 | 1.30–1.56 | 7.25 ± 2.77 | 6.21 ± 2.56 | 1.17 ± 0.42 | 1.00–1.36 |
| Urinary excretion | | | | | | | | |
| (%) total | 22.2 ± 11.0 | 23.0 ± 12.9 | NC | NC | 1.1 ± 0.5 | 0.9 ± 0.4 | NC | NC |

[a]Corrected for differences in actual administered oral (2.3 mg $m^{-2}$) and i.v. dose (1.5 mg $m^{-2}$); NC, not calculated because of carry-over; ND, not detectable for all patients.

 *© 1999 Cancer Research Campaign*

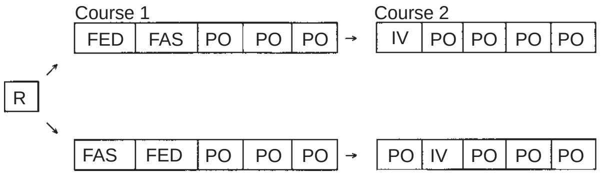

**Figure 2** Schematic representation of the 2 × 2 cross-over design of the study. The symbols represent: R, randomization; FED, fed conditions; FAS, fasted conditions; p.o., oral administration; i.v., intravenous administration. Course 2 commenced 21 days after day 1 of course 1

topotecan and *N*-desmethyltopotecan urine levels as the total of their lactone plus ring-opened carboxylate forms, 1000 μL of urine were diluted in methanol to a final volume of 25.0 ml. After mixing, the solution was acidified with 25 mmol $l^{-1}$ phosphoric acid (50:50 v/v). Then, 25–50 μl of the diluted plasma and urine samples were injected onto the HPLC column (Zorbax SB-$C_{18}$ analytical column, 3.5 μm particle size, 4.6 × 150 mm internal diameter; Rockland Technologies Inc.). The mobile phase was composed of 10 mmol $l^{-1}$ citric acid buffer (adjusted to pH 3.0 with 20 mmol $l^{-1}$ di-sodium hydrogen phosphate) and methanol (75:25 v/v). The operating temperature was 34°C and the flow-rate was 1.0 ml $min^{-1}$. The HPLC system consisted of a Model Spectra System P1000 solvent delivery system (Thermo Separation Products (TSP), Fremont, CA, USA), a Model Spectra AS3000 autosampler (TSP), a Model 7980 thermo regulator (Jones Chromatography Inc., Lakewood, CO, USA) and a Model FP920 fluorescence detector (Jasco International Co. Ltd., Tokyo, Japan). The column eluate was monitored by fluorescence with the excitation wavelength set at 380 nm and the emission wavelength set at 527 nm. Chromatographic data processing was performed with a Model SP4600 DataJet integrator (TSP) coupled to a PC1000 data system and the spreadsheet software package Lotus 1–2–3 (Version 3.4; Lotus; Cambridge, MA, USA). Calibration curves were calculated by least squares linear regression analysis with weight factor $1/x^2$, where x is the analyte concentration. Using 100 μL of plasma the lower limit of quantification for both topotecan and its *N*-desmethyl metabolite was 0.1 ng $ml^{-1}$. Linear responses in the analyte peak area were observed for topotecan concentrations ranging from 0.1 to 50 ng $ml^{-1}$ and for *N*-desmethyltopotecan concentrations ranging from 0.1 to 2.5 ng $ml^{-1}$. Using 1000 μl of urine, the lower limit of quantification was set at 25.0 ng $ml^{-1}$ and 2.5 ng $ml^{-1}$ for topotecan and *N*-desmethyltopotecan respectively. Linear responses in the analyte peak area were observed for topotecan concentrations ranging from 25.0 to 1000.0 ng $ml^{-1}$ and for *N*-desmethyltopotecan concentrations ranging from 2.5 to 100.0 ng $ml^{-1}$. The deviation from the nominal concentration for all tested concentrations was equal to or less than 6%. Within-run and between-run precisions were less than 10%, and average accuracies were between 90 and 110%. Cross-validation of both methods used in this study showed no significant differences (<15%) in topotecan concentration.

### Pharmacokinetic analysis

Pharmacokinetic parameters were obtained using a model-independent approach. For each individual, the maximum drug concentration ($C_{max}$) and time to maximum drug concentration ($t_{max}$) were generated directly from the experimental data. The area under the plasma-concentration-time curve ($AUC_{0-\infty}$) was estimated by the linear–logarithmic trapezoid rule up to the last measured time point ($AUC_{0-t}$) with extrapolation to infinity using the terminal rate constant k. Of each individual plasma concentration versus time profile the data which represented the terminal part of the profile were visually selected and k was estimated by linear regression of the logarithm of the concentrations on sampling time. The apparent terminal half-life ($t_{1/2}$) was calculated as 0.693/k. The relative bioavailability was calculated as the ratio of the $AUC_{0-\infty}$ under fasted and fed conditions. The absolute bioavailability (F) was calculated as the ratio of the $AUC_{0-\infty}$ after oral and i.v. administration corrected for differences in actual administered dose. The percentage of the administered dose recovered in the urine ($U_{excr}$) was calculated as the amount excreted in the urine divided by the actual administered dose *100%, and was corrected for F following oral dosing.

### Statistical analysis

The commercially available software package SPSS® (Statistical Product and Service Solutions, version 6.1 for Windows, Chicago, IL, USA) was used for statistical analysis. Each treatment course represented a two-period cross-over design. The effect of food co-administration was assessed using three parameters namely $C_{max}$, $t_{max}$ and $AUC_{0-\infty}$. For the determination of the absolute bioavailability, $AUC_{0-\infty}$ and $t_{1/2}$ were examined. Pre-dose blood samples on day 2 were checked for any carry-over of drug or metabolite. Prior to statistical analysis a test for carry-over effects between treatments was performed. The statistical analysis employed an analysis of variance (ANOVA) model with effects due to subject, sequence, period and regimen (i.e. fasted or fed, i.v. or oral). The parameters of $C_{max}$, AUC and $t_{1/2}$ were logarithmically transformed; this ensured additivity of the model effects and brought the distribution of the data closer to a normal distribution. The null hypothesis of no difference between the two conditions was tested at a 5% level of significance. Classical 90% confidence intervals (CI) were calculated for the target parameters. According to the currently accepted criteria for assessing bioequivalence (Rabst et al, 1990), the mean log transformed parameters under experimental conditions (i.e. fasted) should be within 80–125% of the parameters under reference conditions (i.e. fed). Due to periodic sampling $C_{max}$ is inherently more variable than the AUC, and a wider acceptance range may be used. To test for differences in $t_{max}$ under fasted and fed conditions the non-parametric Wilcoxon matched-pairs signed-rank test was used. Data are presented as their geometric means ± standard deviation (s.d.).

### RESULTS

### Patients and treatment

Eighteen patients (seven males and 11 females) were enrolled in the study. The median age was 58 years (range 40–75). The median performance score was 1 (range 0–2). Primary tumour types included ovarian (nine), colorectal (six), stomach (two) and non-Hodgkin's lymphoma (one). All patients but one had received previous chemotherapeutic treatment (median 1 regimen, range 0–7). Seven patients used no concomitant medication, the other patients used one or a combination of the following drugs: acetaminophen, codeine, temazepam, oxazepam, metoclopramide, magnesium oxide, granisetron, lactulose, triamterene/hydrochlorothiazide. Overall, the capsules were well-tolerated. The principal toxicity was myelosup-

© 1999 Cancer Research Campaign

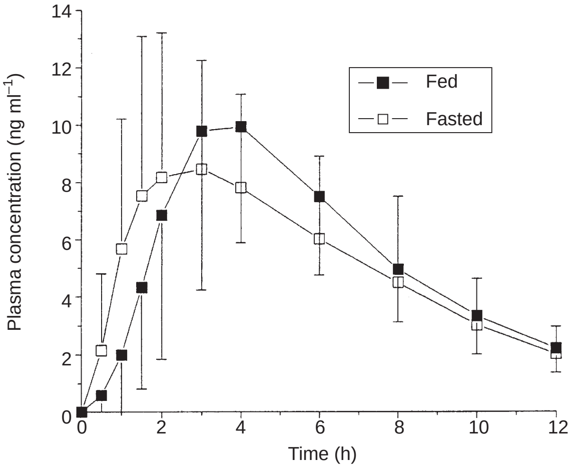

**Figure 3** Mean (± s.d.) plasma concentration-time curves of topotecan as the total of the lactone and hydroxy acid forms after oral administration with (■) or without food (□)

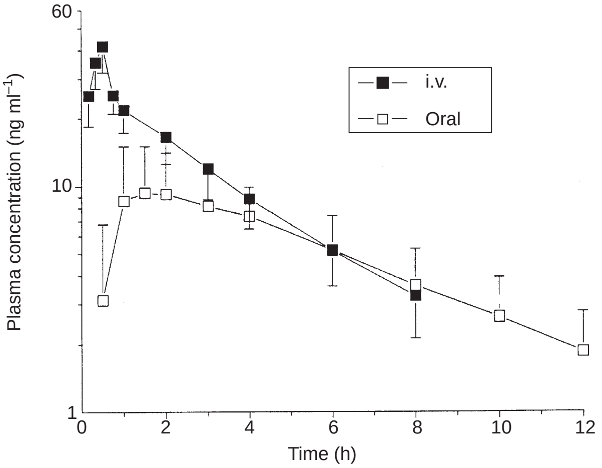

**Figure 4** Mean (± s.d.) plasma concentration-time curves of topotecan as the total of the lactone and hydroxy acid forms after oral (□) and i.v. administration (■). The oral dose was 2.3 mg $m^{-2}$, the i.v. dose was 1.5 mg $m^{-2}$ administered as a 30-min infusion

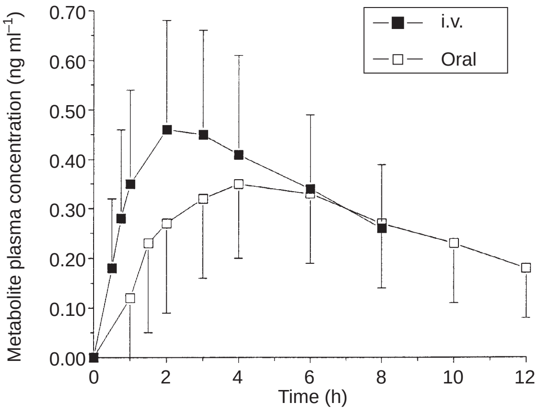

**Figure 5** Mean (± s.d.) plasma concentration-time curves of *N*-desmethyltopotecan as the total of the lactone and hydroxy acid forms after oral (□) and i.v. administration (■) of topotecan pression, primarily neutropenia. Three patients needed dose reductions to 1.9 and 1.25 mg $m^{-2}$ for oral and i.v. topotecan, respectively, in their second treatment course because of NCI CTC grade 4 neutropenia and thrombocytopenia after the first course.

### Pharmacokinetic analysis

Mean plasma concentration–time curves of topotecan as the total of the lactone and carboxylate forms after oral administration with or without food are depicted in Figure 3. Mean pharmacokinetic parameters are summarized in Table 1. One patient was not evaluable because co-medication (triamterene) caused interfering peaks in the HPLC assay. In some patients there were measurable topotecan concentrations at the pre-dose time point for day 2 of both courses. Although the concentrations were very low (mean 0.43 ± 0.24 ng $ml^{-1}$ for total topotecan), this constitutes carry-over. Therefore, an ANOVA model that included a sequence test for carry-over effects was used; the statistical test revealed no significant carry-over effects. After i.v. administration at least 80% of the $AUC_{0-\infty}$ was accounted for by the observed data, which indicates that blood sampling was continued for a long enough period after dosing. However, in both courses the per cent of the $AUC_{0-\infty}$ extrapolated after oral administration was >20% in five patients.

The $AUC_{0-\infty}$ ratio of fasted over high-fat treatment was 0.93 ± 0.23 (90% confidence interval (CI) 0.83–1.03) for topotecan as the total of the lactone and carboxylate forms. Using $AUC_{0-t}$ instead of the $AUC_{0-\infty}$ in the calculations gave the same results: the fasted-to-fed $AUC_{0-t}$ ratio was 0.93 ± 0.25 (90% CI 0.83–1.04). Maximal plasma concentrations of topotecan were similar after ingestion of the capsules with (mean 10.6 ± 4.4 ng $ml^{-1}$) or without food (9.2 ± 4.1 ng $ml^{-1}$) ($P$ = 0.130). The time needed to reach maximal plasma levels was significantly prolonged after food intake (median 3.1 h, range 2.8–6.1) compared to fasted conditions (2.0 h, range 1.1–8.1) ($P$ = 0.013).

Mean plasma concentration–time curves of total topotecan and total *N*-desmethyltopotecan after oral and i.v. administration are depicted in Figures 4 and 5. Mean pharmacokinetic parameters are summarized in Table 2. The absolute bioavailability of total topotecan averaged 42 ± 13% (90% CI 37–47%). Using $AUC_{0-t}$ instead of $AUC_{0-\infty}$ resulted in a value for F of 40 ± 14%. The lactone:total topotecan $AUC_{0-\infty}$ ratio was slightly but significantly lower after oral (0.35 ± 0.06) than after i.v. administration (0.38 ± 0.06) ($P$ = 0.002). The correlation coefficient between the oral and i.v. lactone:total ratio was 0.84 ($P$ < 0.001). The apparent terminal half-life was significantly longer after administration of oral topotecan (3.9 ± 1.0 h) than after i.v. administration (2.7 ± 0.4 h) ($P$ < 0.001).

*N*-desmethyltopotecan lactone concentrations were below the detection limit in six patients and in most other patients the elimination phase could not satisfactorily be determined. Therefore, the *N*-desmethyltopotecan lactone AUC was not calculated. For the total metabolite $AUC_{0-\infty}$ the percentage extrapolated was >20% in most patients. The oral:i.v. $AUC_{0-t}$ and $AUC_{0-\infty}$ ratios of *N*-desmethyltopotecan as the total of lactone and carboxylate forms were 71 ± 39% (90% CI 56–87%) and 62 ± 20% (90% CI 54–71%), respectively, corrected for differences in actual administered topotecan dose. Maximal total metabolite plasma levels were reached at 4.0 h (median, range 2.0–6.2) after ingestion of

© 1999 Cancer Research Campaign topotecan capsules and at 2.1 h (median, range 2.0–3.1) after the start of the infusion ($P$ = 0.002). *N*-desmethyltopotecan had a long apparent terminal half-life compared to that of the parent compound, being 7.3 ± 2.8 h and 6.2 ± 2.6 h after oral and i.v. topotecan administration, respectively. The metabolic ratio, defined as the metabolite:topotecan AUC ratio, was significantly higher after oral (6.9 ± 4.0%) than after i.v. administration (4.5 ± 2.5%) ($P < 0.001$).

The urinary excretion of total topotecan was 22.2 ± 11.0% of the oral dose corrected for F and 23.0 ± 12.9% of the i.v. dose. However, topotecan urinary recovery is incomplete within 24 h after dosing and, consequently, the excretion data were confounded by carry-over effects between treatments. For the *N*-desmethyl metabolite the urinary excretion was 1.1 ± 0.5% and 0.9 ± 0.4% of the administered topotecan dose after oral and i.v. administration respectively.

No relationships were noted between patient characteristics, including age, gender, tumour site, renal and hepatic function tests, and the bioavailability and metabolism of topotecan. However, the limited number of patients may obscure such relationships.

### DISCUSSION

The high anti-tumour activity in vitro and in vivo with prolonged exposure to topotecan (Burris et al, 1988; Supko et al, 1992; Houghton et al, 1995) and the inconvenience for the patient associated with daily intravenous administration for prolonged periods encouraged the development of an oral formulation. In previous studies, bioavailability of the i.v. formulation of topotecan administered orally was 30–44% with moderate interpatient variability (Eckardt et al, 1995; Schellens et al, 1996). A phase I study of a gelatin capsule formulation of topotecan has determined the MTD to be 2.3 mg $m^{-2}$ for the daily times five dosing schedule (Gerrits et al, 1998). Dose-limiting toxicity (DLT) was NCI CTC grade 4 neutropenia, which is consistent with that of the i.v. formulation. The regimen was well-tolerated. Phase II studies are currently being conducted with the oral capsule formulation in several tumour types including ovarian, lung, and breast carcinoma. Neutropenia is the principal toxicity. Other side-effects including nausea/vomiting, diarrhea, fatigue and alopecia occur, but to a lesser extent.

The present study is an important study in the clinical development of oral topotecan and the first to report on the absolute and relative (i.e. impact of diet) availability of the gelatin capsule formulation. Topotecan undergoes reversible hydrolysis; a free, non-enzymatic, pH-dependent equilibrium exists between the active lactone and an inactive carboxylate form. This potential for conversion from carboxylate to lactone suggests that cytotoxic activity can be present regardless of what proportion of topotecan is in the open-ring form in the extracellular space. According to the Food and Drug Administration (FDA) definition of bioavailability, the ‘active moiety’ should include all components of a reversible metabolism (Marzo et al, 1995). Therefore, total (lactone plus carboxylate forms) topotecan levels were used to determine the relative and absolute bioavailability of the drug.

The statistical procedure for testing carry-over effects indicated that the washout period of 24 h between treatments was long enough; however, measurable topotecan concentrations were observed in pre-dose blood samples on day 2. Regulatory authorities require bioequivalence to be assessed on extrapolated AUC and not on $AUC_{0\text{-}t}$. This extrapolation is considered acceptable when the area added is ≤20% of the whole extrapolated AUC (CPMP, 1992; Marzo et al, 1995). In the current study, the analysis of the $AUC_{0\text{-}\infty}$ after oral administration involved some AUC values that were more than 20% extrapolated, but the effect on the calculation of the bioavailability was minimal as indicated by the similar results obtained using either $AUC_{0\text{-}\infty}$ or $AUC_{0\text{-}t}$.

The AUCs and maximal plasma concentrations of topotecan were similar after ingestion of the capsules with or without food. In addition, the apparent terminal half-life was not affected. For these parameters the two treatment conditions fulfilled the bioequivalence criteria. The time needed to reach maximal plasma levels was significantly prolonged after food intake compared to fasted conditions. Food, particularly fat, retards gastric emptying and so decreases the rate of drug absorption. Summarizing, coadministration of topotecan capsules with food leads to a small decrease in absorption rate but does not affect the overall extent of absorption. Therefore, administration of topotecan with or without food can be judged therapeutically equivalent since the relationship between absorption rate and toxicity is unclear and nothing of clinical significance was observed in the results.

The absolute bioavailability of topotecan averaged 42%, which is in good agreement with previous reports (Eckardt et al, 1995; Schellens et al, 1996). The bioavailability of topotecan calculated using only the lactone data was 38%. This indicates that the reversible hydrolysis of topotecan has little or no effect on the bioavailability estimate for this drug, which is not surprising considering the fact that it is a reversible chemical rather than a reversible enzymatic reaction that occurs. The low oral bioavailability of topotecan may be explained by presystemic, i.e. in the gut, ring-opening of topotecan lactone yielding substantial amounts of the carboxylate form. Only the lactone form of topotecan is assumed to be sufficiently lipophilic to traverse membranes in the gastrointestinal tract. This is supported by studies in dogs that demonstrated that approximately 2.5% of an absorbed oral dose of topotecan enters the blood circulation as the open-ring form, which suggests that the open-ring form is poorly absorbed (Davies et al, 1995). In our study, the small but statistically significant difference in lactone:total AUC ratio after oral and i.v. administration could therefore result from a small fraction of the dose absorbed as the carboxylate form.

Oral administration of topotecan was accompanied by a substantially increased interpatient variability in systemic exposure; the coefficient of variation of the $AUC_{0\text{-}\infty}$ was 44% after oral administration compared to 25% after i.v. administration. The apparent terminal half-life was significantly longer after oral administration of topotecan than after i.v. administration. The absorption half-life of topotecan must therefore be longer than the elimination half-life. This indicates that absorption is the rate-limiting step in the elimination of the drug. Metabolism of topotecan to a recently identified *N*-desmethylated metabolite represented a minor metabolic pathway (Herben et al, 1997; Rosing et al, 1997). Although *N*-desmethyltopotecan has slightly less topoisomerase I-inhibiting activity than the parent compound (Johnson and Wood, personal communication), it is unlikely to be of therapeutical importance. *N*-desmethyltopotecan lactone concentrations were below the detection limit in most patients. The percentage of the total *N*-desmethyl topotecan $AUC_{0\text{-}\infty}$ estimated using extrapolation was >20% in most patients, which indicates that for this metabolite with a relatively long half-life, blood sampling over 12 h post-topotecan administration was too short. The metabolic ratio ($AUC_{0\text{-}\infty}$ metabolite:topotecan) was only 6.9%

© 1999 Cancer Research Campaign 

and 4.5% after oral and i.v. administration respectively. The higher metabolic ratio after oral compared to i.v. administration indicates that *N*-desmethyltopotecan is a first-pass metabolite. The inclusion of the metabolite $AUC_{0-\infty}$ in the calculation of the absolute bioavailability did not change this value significantly (43% instead of 42%).

In conclusion, topotecan demonstrates suitable bioavailability for oral treatment. Co-administration of the topotecan gelatin capsules with a high-fat breakfast leads to a small decrease in absorption rate but does not affect the extent of absorption.

### ACKNOWLEDGEMENTS

This work was financially supported by a grant from SmithKline Beecham Pharmaceuticals, King of Prussia, PA, USA.

### REFERENCES

Ajani JA, Welch SR, Raber MN, Fields WS and Krakoff IH (1990) Comprehensive criteria for assessing therapy-induced toxicity. *Cancer Invest* **8**: 147–159

Burris HA III, Hanauske AR, Johnson RK, Marshall MH, Kuhn JG, Hilsenbeck SG and Von Hoff DD (1992) Activity of topotecan, a new topoisomerase I inhibitor, against human tumor colony-forming units. *J Natl Cancer Inst* **84**: 1816–1820

Creemers GJ, Gerrits CJH, Eckardt JR, Schellens JHM, Burris HA, Planting AST, Rodriguez GI, Loos WJ, Hudson I, Broom C, Verweij J and Von Hoff DD (1997) Phase I and pharmacologic study of oral topotecan administered twice daily for 21 days to adult patients with solid tumors. *J Clin Oncol* **15**: 1087–1093

Committee for Proprietary Medicinal Products (1992) *Note for Guidance: Investigation of Bioavailability and Bioequivalence (III/54/89-EN)*. CPMP: Brussels

Davies BE, Minthorn EA, Dennis M, Beijnen JH and Rosing H (1995) The pharmacokinetics of topotecan and its carboxylate form following separate intravenous administration to the dog. *Eur J Cancer* **31A**: S28

Eckardt J, Burris H, Rizzo J, Fields S, Rodrigues G, DelaCruz P, Hodges S, Von Hoff and Kuhn J (1995) A phase I safety and bioavailability study of oral topotecan. *Eur J Cancer* **31A**: S193

Gerrits CJH, Burris H, Schellens JHM, Planting AST, Van der Burg MEL, Rodrigues GI, Van Beurden V, Loos WJ, Hudson I, Fields S, Verweij J and Von Hoff DD (1998) Five days of oral topotecan (Hycamtin®), a phase I and pharmacological study in adult patients with solid tumours. *Eur J Cancer* **7**: 34

Herben VMM, Ten Bokkel Huinink WW, Dubbelman AC, Mandjes IAM, Groot Y, Van Zomeren DM and Beijnen JH (1997) Phase I and pharmacologic study of sequential intravenous topotecan and oral etoposide. *Br J Cancer* **76**: 1500–1508

Hertzberg RP, Caranfa MJ, Holden KG, Jakas DR, Gallagher G, Mattern MR, Mong SM, Bartus JO, Johnson RK and Kingsbury WD (1989) Modification of the hydroxy lactone ring of camptothecin: inhibition of mammalian topoisomerase I and biological activity. *J Med Chem* **32**: 715–720

Houghton PJ, Cheshire PJ, Hallman JD II, Lutz L, Friedman HS, Danks MK and Houghton JA (1995) Efficacy of topoisomerase I inhibitors, topotecan and irinotecan, administered at low dose levels in protracted schedules to mice bearing xenografts of human tumors. *Cancer Chemother Pharmacol* **36**: 393–403

Hsiang Y-H and Liu LF (1988) Identification of mammalian DNA topoisomerase I as an intracellular target of the anticancer drug camptothecin. *Cancer Res* **48**: 1722–1726

Hsiang Y-H, Liu LF, Wall ME, Wani MC, Nicholas AW, Manikumar G, Kirschenbaum S, Silber R and Potmesil M (1989) DNA topoisomerase I-mediated DNA cleavage and cytotoxicity of camptothecin analogs. *Cancer Res* **49**: 4385–4389

Marzo A and Balant LP (1995) Bioequivalence. An updated reappraisal addressed to applications of interchangeable multi-source pharmaceutical products. *Arzneim-Forsch/Drug Res* **45**: 109–115

Pabst G and Jaeger H (1990) Review of methods and criteria for the evaluation of bioequivalence studies. *Eur J Cancer* **38**: 5–10

Pommier Y, Jaxel C, Kerrigan D and Kohn KW (1991) Structure–activity relationship of topoisomerase I inhibition by camptothecin derivatives: evidence for the existence of a ternary complex. In *DNA Topoisomerases in Cancer*, Potmesil M and Kohn KW (eds), pp. 121–132. Oxford University Press: New York

Rosing H, Doyle E, Davies BE and Beijnen JH (1995) High-performance liquid chromatographic determination of the novel antitumour drug topotecan and topotecan as the total of the lactone plus carboxylate forms, in human plasma. *J Chrom B* **668**: 107–111

Rosing H, Herben VMM, Van Gortel-van Zomeren DM, Hop E, Kettenes-van den Bosch JJ, Ten Bokkel Huinink WW and Beijnen JH (1997) Isolation and structural confirmation of N-desmethyl topotecan, a metabolite of topotecan. *Cancer Chemother Pharmacol* **39**: 498–504

Schellens JHM, Creemers GJ, Beijnen JH, Rosing H, De Boer-Dennert M, McDonald M, Davies B and Verweij J (1996) Bioavailability and pharmacokinetics of oral topotecan: a new topoisomerase I inhibitor. *Br J Cancer* **73**: 1268–1271

Supko JG, Plowman J, Dyker DJ and Zaharko DS (1992) Relationship between the schedule dependence of 9-amino-20(S)-camptothecin (AC; NSC 603071) antitumor activity in mice and its plasma pharmacokinetics. *Proc Am Assoc Cancer Res* **33**: 432

Underberg WJM, Goossen RMJ, Smith BR and Beijnen JH (1990) Equilibrium kinetics of the new experimental anti-tumour compound SK&F 104864-A in aqueous solution. *J Pharm Biomed Anal* **8**: 681–683

 © 1999 Cancer Research Campaign
